# Silicon Nitride-Based Composites with the Addition of CNTs—A Review of Recent Progress, Challenges, and Future Prospects

**DOI:** 10.3390/ma13122799

**Published:** 2020-06-21

**Authors:** Awais Qadir, Péter Pinke, Ján Dusza

**Affiliations:** 1 Doctoral School of Materials Science & Technologies, Óbuda University, 1034 Budapest, Hungary; jdusza@saske.sk; 2 Institute of Materials & Manufacturing Sciences, Donat Banki Faculty of Mechanical & Safety Engineering, Óbuda University, 1081 Budapest, Hungary; pinke.peter@bgk.uni-obuda.hu; 3 Institute of Materials Research, Slovak Academy of Sciences, Košice 04001, Slovakia

**Keywords:** Si_3_N_4_, carbon nanotubes (CNTs), MWCNTs, mechanical properties, CMCs

## Abstract

In this overview, the results published to date concerning the development, processing, microstructure characteristics, and properties of silicon nitride/carbon nanotube (Si_3_N_4_ + CNTs) composites are summarized. The influence of the different processing routes on the microstructure development of the Si_3_N_4_ + CNTs is discussed. The effects of the CNTs addition on the mechanical properties—hardness, bending strength and fracture toughness—and tribological characteristics—wear rate and coefficient of friction—are summarized. The characteristic defects, fracture origins, toughening and damage mechanisms occurring during the testing are described. The influence of the CNTs’ addition on the thermal and functional properties of the composites is discussed as well. New trends in the development of these composites with significant potential for future applications are outlined.

## 1. Introduction

In 1859, the synthesis of Si_3_N_4_ was reported for the first time by Sainte-Claire Deville and Wohler [1]. Si_3_N_4_ is a non-oxide ceramic material and is considered as an advanced ceramic material because of its exceptional properties in different engineering applications. Silicon nitride-based materials have been used as cutting tools, bearings, sealings, parts of gas turbines, engines, etc., due to their exceptional combination of mechanical properties as flexural strength, hardness, resistance to oxidation, tribological and thermal properties [2,3,4,5,6,7,8].

However, silicon nitride, as a structural ceramic material with a number of excellent properties, also exhibits some negative properties, such as brittleness, low flaw tolerance, a limited number of slip systems and low reliability, which limit its wider applications [3,8]. During the last few decades, an addition of a second phase to the silicon nitride matrix or the development of in situ reinforced Si_3_N_4_ was proposed to overcome the low flaw tolerance and reliability of the system. An improvement was achieved to some level, but many problems have arisen as well. With the addition of second phases in the form of particles, whiskers or fibers, the major problems are the inhomogeneous dispersion-agglomeration, pores and impurities [9,10,11].

Recent advances in nanomaterials research and development opened up new opportunities to tailor the ceramic structures at nanometric scale, and to develop new classes of silicon nitride-based ceramics with improved mechanical properties and functionalities.

After the discovery of carbon nanotubes (CNTs) by Ijima [12], a new horizon of research arose in the materials science field. Since its discovery, CNT-based materials have been investigated widely, a high number of ceramic-based nanocomposites have been developed and several applications were proposed to be applied in the electrical, tribological and load-bearing sectors [13,14,15,16,17,18,19,20,21,22,23,24]. The exceptional mechanical, electrical, thermal and multifunctional properties of CNTs made it a potential candidate for reinforcement to achieve better properties of the composite. Four factors are important in measuring the efficiency of CNTs as a reinforcement in a ceramic matrix: (a) intrinsic mechanical properties, (b) load transfer efficiency, (c) dispersion level and (d) interfacial bonding between the CNTs and matrix grains. To achieve optimum mechanical, electrical or tribological properties of densified ceramics, different ceramic composites have been developed with the addition of CNTs and graphene [13,14,15,16,17,18]. The aim of these investigations was to develop ceramic-based composites with improved fracture toughness, increased thermal and electrical conductivity and increased thermal shock resistance and wear characteristics.

Up to the date of writing this review, we searched through a number of publications on this topic. Overall, 696 documents were retrieved when the topics “CNT” and “Ceramic” were searched on the Web of Science (WOS). When we refined the search with the keywords “CNT” and “Si_3_N_4_”, 51 documents were retrieved. A number of publications cannot be properly considered as Si_3_N_4_ + CNT composites because some publications are not relevant to our topic. We carefully considered the relevant publications to write this review paper. To the best of our knowledge, there is no review paper on CNT-reinforced silicon nitride composites, and therefore our main focus is to highlight the advancements in this topic. It is difficult to compare the properties of composites produced and investigated by different authors, because each author used different processing techniques, parameters and testing methods for Si_3_N_4_ + CNT composite.

This review discusses the different types of CNT used as additives to the silicon nitride matrix, the main processing routes and microstructure characterization techniques and the influence of the processing routes on the microstructure characteristics during the development of the Si_3_N_4_ + CNT composites. Significant attention is devoted to the explanation of the effect of the CNTs’ addition on the mechanical properties-hardness, bending strength and fracture toughness and tribological characteristics, including the wear rate and the coefficient of friction. In the end, the influence of the CNTs’ addition on the thermal and functional properties of the composites is discussed, and future progress expected in the development of Si_3_N_4_ + CNT composites is predicted.

## 2. Silicon Nitride (Si_3_N_4_)

**Figure 1:**
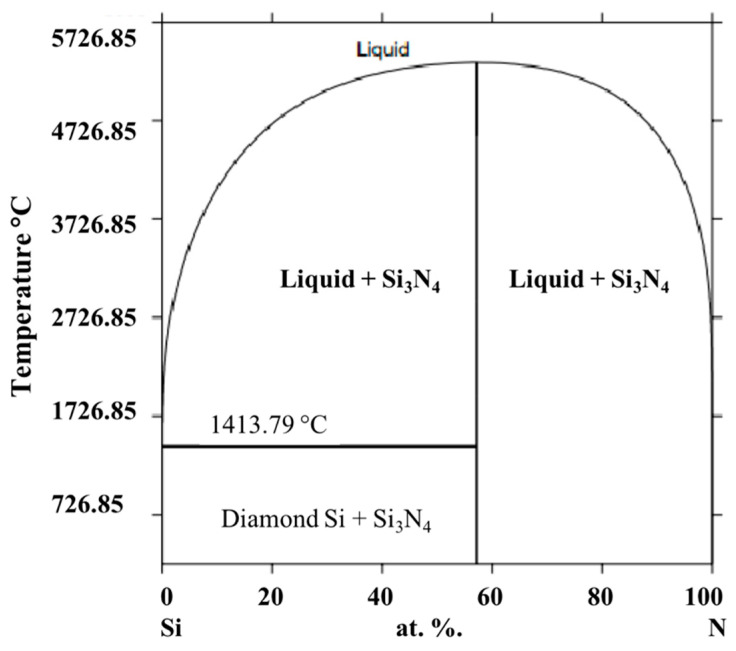
Calculated phase diagram of Si-N system based on [25].

Silicon nitride (Si_3_N_4_) is classified as an advanced structural ceramic with a high melting point, high hardness, and is relatively chemical inert. Si_3_N_4_ is the stoichiometric compound in the Si–N binary system [25]. Other silicon nitrides (Si_2_N_3_ [26], SiN [27], Si_3_N [28], Si(N_3_)_4_ in this Si–N binary system have been reported, but their existence was considered doubtful. The calculated phase diagram of Si–N system is given in Figure 1.

It has three crystallographic structures at room temperature, which are named as α, β and Υ. α and β are most common crystallographic phases of silicon nitride and have technological applications in advanced ceramics [2,29,30]. Every crystallographic structure has its own characteristics whose presence influences the final properties of the silicon nitride composite. The Υ phase of silicon nitride has a cubic structure, and therefore it is the hardest phase, with a value of up to 35 GPa, and is not widely used for structural applications [30,31]. The β phase has an elongated hexagonal structure and high toughness. The α phase has a trigonal structure and is harder than the β phase [31].

## 3. Carbon Nanotubes (CNTs)

**Figure 2:**
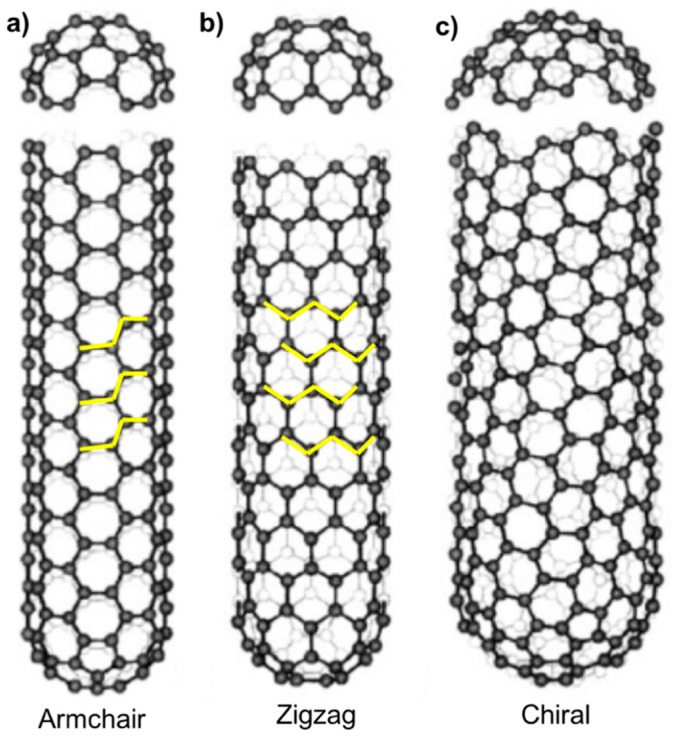
Three types of carbon nanotubes (CNTs): (**a**) armchair (n, m) = (5, 5); (**b**) zigzag (n, m) = (9, 0); (**c**) chiral (n, m) = (10, 5) [32]. Reproduced from Ref. [32] with permission from Taylor and Francis.

During the production of carbon C60 and fullerene by arc evaporation of graphite, Ijima examined the deposited carbon layer on the graphite by high resolution transmission electron microscope (HRTEM) [12]. He discovered a new form of carbon which consisted of a graphene cylinderical tube with 10 nm diameter and the end-cap was like a fullerene structure. This new form of carbon was named a carbon nanotube (CNT) after its physical appearance. A CNT has three basic orientations (Figure 2) [32]:

Armchair orientation: Graphene cylinder along a five-fold axis with a fullerene-like cap at the end (Figure 2a).

Zigzag orientation: Graphene cylinder along a three-fold axis with a fullerene-like cap at the end (Figure 2b).

Chiral orientation: Graphene cylinder along a helical arrangement with a fullerene-like cap at the end (Figure 2c).

Carbon nanotubes (CNTs) are classified into two groups: multi-walled carbon nanotubes (MWCNTs) and single-walled carbon nanotubes (SWCNTs) [33]. MWCNTs consist of multiple concentric graphene cylinders, while SWCNTs comprise a single layer of a graphene cylinder. The diameter of a CNT ranges between 1 and 50 nm and the length ranges from a few nm to a few µm [34,35,36,37]. A CNT has a tensile strength 10 times greater than that of steel and its stiffness is 15 times higher than that of steel (Table 1). The comparison of properties of different materials is given in Table 1.

CNTs can be produced in two main ways:

(i) Arc Evaporation Method: A 50 Ampere current is applied between graphite electrodes to evaporate the graphite and this is done in a helium environment. CNTs are condensed at the cathode. Ijima also used this method to produce CNTs [12]. SWCNTs can be produced by this method with addition of Ni and Co at graphite anode electrode [38].
(ii) Catalytic Method: CNTs are produced by the decomposition of hydrocarbons over the metallic catalysts (Fe, Co, Ni) [21,39]. This method has the one disadvantage that CNTs are produced with lattice defects more than that of the arc evaporation method. These defects can be reduced by heat treatment after the production [40].

## 4. Processing of Si_3_N_4_ + CNT Composites

**Figure 3:**
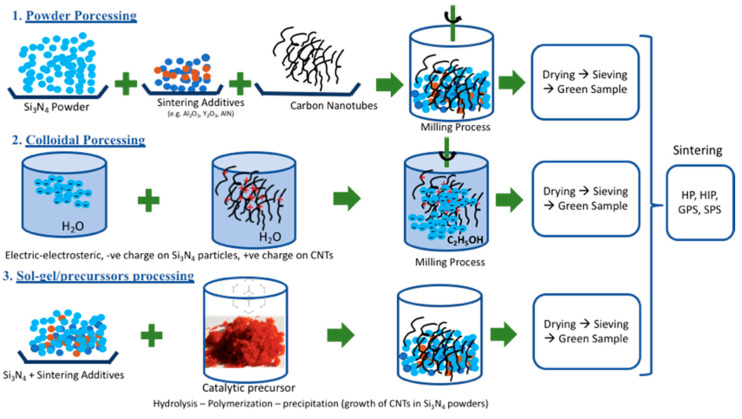
Illustration of the main processing routes used for the processing of Si_3_N_4_ + CNT composites.

It is difficult to sinter Si_3_N_4_ to achieve full density due to the presence of covalent bonds between Si and N atoms. The processing of CNTs based Si_3_N_4_ composites is even more difficult because of the integration of a reinforcement phase at the nanometric scale, therefore the processing routes have to be optimized before manufacturing the Si_3_N_4_ + CNT composites. The main processing routes of a Si_3_N_4_ + CNT composite are schematically illustrated in Figure 3, in which the powder preparation phase before sintering can be in the form of:

(i) Powder processing
(ii) Colloidal Processing
(iii) Sol-gel/precursor (in situ growth of CNTs).

After the processing route of starting powders, several techniques (i.e., hot pressing, hot isostatic pressing, gas pressure sintering, and spark plasma sintering) have been applied to densify the silicon nitride-based powders. Many sintering additives have been used with the aim of improving the sintering process [2,41]. In addition to the positive influence, the sintering additives have a negative aspect as well; they segregate at the grain boundaries and have a negative effect on the high-temperature mechanical properties.

### 4.1. Sintering Additives

Due to covalent bonding and low diffusivity, Si_3_N_4_ cannot be fully densified by solid-state sintering without any additives. Addition of sintering additives introduces a so-called liquid-phase sintering process which results in higher densification [2]. In the case of CNT-reinforced silicon nitride composites, a wide range of sintering additives of metal oxides or non-oxides were used. So far in the literature, these additives (TiO_2_, Y_2_O_3_, Al_2_O_3_, MgO, SiO_2_, AlN, HfO_2_ and ZrO_2_) were reported as sintering additives for the fabrication of CNT-reinforced silicon nitride composites [13,15,18,42,43,44]. The most often used sintering additives were Y_2_O_3_, Al_2_O_3_ and ZrO_2_. Recently, Matsuoka et al. [13] added HfO_2_ to Y_2_O_3_-Al_2_O_3_-AlN additives to prevent the CNTs from reacting and disappearing from the composite. They reported that the addition of HfO_2_ resulted in higher electrical conductivity (~102 S/m) and higher bending strength (~1086 MPa). It was also observed that the addition of AlN enhanced the electrical conductivity of the Si_3_N_4_ + CNT composites [18]. According to the published results, the optimized composition and amount of sintering additives is very important for processing high-density composites with good mechanical and functional properties.

### 4.2. Milling Process

The degree of dispersion of carbon nanotubes in the silicon nitride matrix significantly affects the final properties of the composite. To achieve excellent properties of CNT-reinforced silicon nitride composites, fully densified composites with uniformly dispersed, undamaged, and un-agglomerated incorporation of CNTs are inevitable. One of the major issues during the integration of carbon nanotubes in the silicon nitride matrix is the difficulty in obtaining uniform dispersion of the nano-fillers owing to their tendency of agglomeration due to van der Waals forces. Agglomerates occur due to high surface area and high aspect ratio of the carbon nanotubes, which critically affects the mechanical properties of composites.

Several researchers emphasized the improvement in the milling process and surface treatment of CNTs before the sintering process, which enhances the uniform dispersion of CNTs in the matrix, and eventually, uniform dispersal improves the density of the sintered composites [45,46].

**Figure 4:**
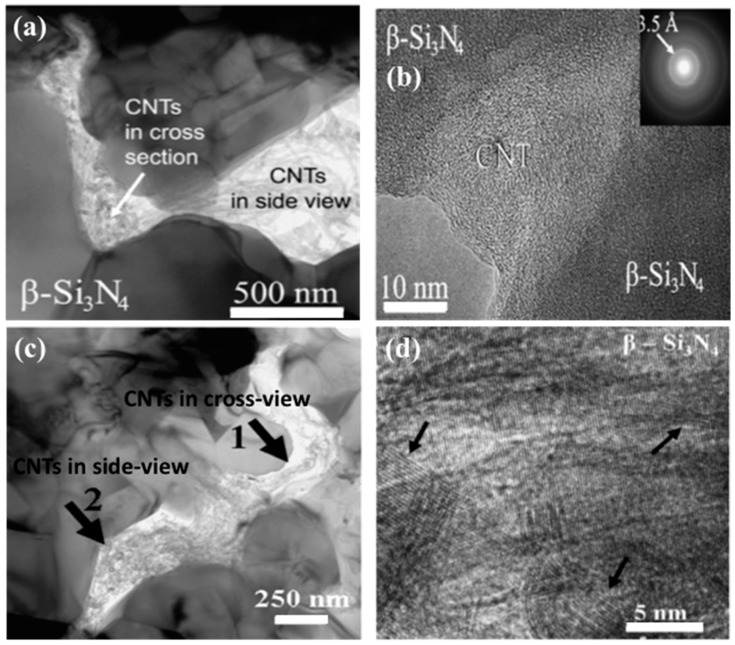
CNT-reinforced Si_3_N_4_ composites: (**a**) Si_3_N_4_ with 1 wt.% CNTs prepared by ball milling, CNTs are located in porosities enclosed with β-Si_3_N_4_ grains; (**b**) HREM revealed that the CNTs were embedded between two β-Si_3_N_4_ grains and electron diffraction confirmed the presence of CNTs with good contact between nanotubes and the surface of silicon nitride grains; (**c**) TEM image of Si_3_N_4_ + CNT composite prepared by attrition milling. CNTs are located in porosities between Si_3_N_4_ grains. (**d**) HREM image shows the silicon nitride-CNTs interfaces [45]. Reproduced from Ref. [45] with permission from Elsevier.

Balazsi et al. [45] prepared Si_3_N_4_ with (1 wt.% and 3 wt.%) MWCNTs by various milling techniques (ball and high attritor milling) and densified by hot isostatic pressing (HIP). Based on transmission electron microscopic (TEM) and high resolution electron microscopic (HREM) results, CNTs were located in porosities and intergranular places in both cases of milling (Figure 4). However, attrition milling was found to be more efficient than that of ball milling in regard to β–Si_3_N_4_ crystallites and pore sizes. β–Si_3_N_4_ crystallite grains ~1 µm and pore size ~500 nm were measured in the case of ball milling. β–Si_3_N_4_ crystallite grains ~300 nm and pore size ~200 nm were measured after sintering in the case of high-efficiency attritor milling.

To achieve an optimum grain size and uniform dispersion of reinforcement in the matrix, the type of milling, time duration of milling, rpm, and type of surfactants are very important. Tapasztó et al. [46] analyzed the effect of milling time on the sintering kinetics of silicon nitride/CNT composites. They found that milling time has a significant influence on the morphology, structure, degradation of carbon fibers, dispersion, phase transformation, and mechanical properties of the resulting composite [46]. Longer milling time reduced the particle size of the starting powder, which enhanced the density and dispersion of CNTs in the matrix, and consequently the mechanical properties were improved [46]. A further increase in milling may damage the carbon nanotubes in the powder and degrade the mechanical properties accordingly. So, the milling time (3–24 h), rpm (4000–6000 rpm), and surfactants (polyethyleneglycol-PEG) must be optimized to achieve optimum mechanical properties of the final composite.

Matsuoka et al. [47] prepared the CNT-reinforced silicon nitride composite with bead milling and used HfO_2_ as a sintering additive. Bead milling was found to be superior to the ball milling in terms of the uniform dispersion of CNTs in the silicon nitride. CNTs’ uniform dispersal promotes the densification, relative density, and strength of the composite. More minor damage of CNTs was found after bead milling than that of ball milling because bead milling applies higher shear stresses, which pulverizes the CNTs [47].

### 4.3. Sintering Routes

A number of sintering techniques have been used for the densification of CNT-reinforced silicon nitride composites by several researchers over the last decade. Hot pressing (HP), hot isostatic pressing (HIP), gas pressure sintering (GPS) and spark plasma sintering, or the combination of GPS and HIP, have been used so far to produce Si_3_N_4_ + CNT composites [45,46,47,48,49,50,51,52,53,54,55,56,57,58,59,60].

During the hot-pressing (HP) technique, mechanical pressure is applied along with high temperature (1500–1800 °C) to densify the powders and the mechanical pressure acts as a driving force to accelerate the rearrangement of the particles. Highly dense silicon nitride composites can be achieved by this process, and it is more effective in improving the mechanical properties than the pressure-less sintering. Pasupuleti et al. [44] produced the monolithic and 1 wt.% CNT-reinforced silicon nitride composite by hot pressing at 1750 °C under 30 MPa pressure for 1 h holding time. They achieved above 99% density of a monolithic silicon nitride composite, which results in high hardness (HV = 15.7 GPa) and flexural strength (1046 MPa). In the case of 1 wt.% CNT-reinforced silicon nitride composite, they achieved >98.7% density with hardness (HV = 15.0 GPa) and flexural strength (996 MPa).

The hot isostatic pressing (HIP) technique has been used widely in fabricating the silicon nitride-based composites. During this technique, an isostatic gas pressure (usually N_2_ gas) is applied to densify the powder in a metal container under a vacuum environment. Usually, the pressure in HIP is about several hundreds of MPa and the optimum temperature range is 1600–1700 °C to achieve the highly dense material. Balázsi et al. [15] and Kovalcikova et al. [48] prepared a CNT-reinforced silicon nitride composite by hot isostatic pressing and achieved high density with better results in mechanical properties.

During gas pressure sintering (GPS), there are two steps involved: in the initial step, the sample is sintered at a high temperature until there are only internal pores. In the second step, gas pressure is applied to eliminate the internal porosity for high densification. This was observed by a few researchers who used the GPS along with hot pressing (HP) or hot isostatic pressing (HIP) to consolidate the Si_3_N_4_ + CNT composites [13,22].

Spark plasma sintering (SPS) is a relatively novel technique where the pulses of electric current are applied to the sample within a conductive die. The large current flow produces the joule heating in the sample in a very short time and densifies the powders. SPS is similar to the hot pressing—the electrical field heats the die and compacts the powders, instead of using indirect heating. During SPS, a shorter sintering time is applied, which restricts the grain growth and can produce composites with a higher density than other sintering techniques. SPS has been applied widely in producing the Si_3_N_4_ + CNT composites [14,49,50,51,52,55]. In the literature, an optimum time range of 3–5 min has been reported to fabricate the silicon nitride-based composites. The type of sintering technique has a significant role in achieving a highly dense composite with mechanical improvement. It is always difficult to compare the results produced with different conditions in different laboratories.

Tapaszto et al. [53] prepared the CNT-reinforced silicon nitride composites by two methods, HIP and SPS. In terms of hardness and stiffness, the SPS technique was found to be better than HIP, but the HIP technique was found to be better in terms of toughness [53]. Samples sintered by SPS contained mainly α–Si_3_N_4_, while samples sintered by HIP consisted of mainly α–Si_3_N_4_. The complete transformation of α to β silicon nitride was observed in samples sintered by HIP [53].

Balazsi et al. [49] developed the Si_3_N_4_–1 wt.% MWCNTs by spark plasma sintering (SPS) with an apparent density of 3.17 g/cm^3^, almost equal to the theoretical density. The same composite Si_3_N_4_–1 wt.% MWCNTs was produced by hot isostatic pressing (HIP) with lower apparent density of 2.5 g/cm^3^ [49]. Highly dense samples were produced by SPS, which results in improved mechanical properties [49]. Here, Balazsi et al. [49] produced the same silicon nitride composite with MWCNTs by two different techniques, SPS and HIP, under the same conditions.

SPS has a better efficiency in producing Si_3_N_4_ + MWCNT composites, despite a lower sintering temperature (1500–1650 °C) and shorter holding time (3–5 min) being applied; the SPS was proven to be a better technique than that of HIP, where the sintering temperature was 1700 and holding time was 3 h. Higher apparent density (up to 3.24 g/cm^3^) was achieved, which led to better mechanical properties, such as toughness (KIC) (up to 6.5 MPa·m^1/2^) and elastic modulus (E) (up to 326.21 GPa) [49].

The amount of porosity plays a detrimental role to many mechanical properties and CNTs are susceptible to inducing porosity in the composite during sintering. Balázsi et al. found that CNTs (0 to 5 wt.%) induced porosity which caused the lowering of the elastic modulus from approximately 260 to 70 GPa [18].

During the sintering process at a high temperature and under high pressure for a long holding time, an interfacial reaction can occur between Si_3_N_4_ and CNTs. The reaction between CNTs and Si_3_N_4_ can produce SiC, which can be a good addition to the composite as a reinforcement for improving the mechanical properties. Ge et al. reported [61] the formation of SiC as a result of the reaction between CNTs and Si_3_N_4_ during the sintering. The equilibrium reaction between carbon and Si_3_N_4_ is given below (Equation (1)) [62,63]:

(1) $${\mathrm{Si}}_{3}N_{4}+3C\;\overset{1510-1550\;℃}{↔}\;3\mathrm{SiC}+2N_{2}$$

Si_3_N_4_ powder particles possess the surface oxygen in the form of the SiO_2_ nanolayer. This oxygen-containing phase reacts with the surface of CNTs at higher temperatures and produces the CO and CO_2_ gases (Equation (2)). The diameter of CNTs might be reduced due to the loss of carbon as a result of surface reaction between C and SiO_2_. Carbon may cause a mass loss in the sintered samples during sintering because of the reduction of SiO_2_. The damage to CNTs during the sintering process was observed by the researchers [15,42].

(2) $$3{\mathrm{SiO}}_{2}\;(l)+6C\;(s)+2N_{2}\;(g)\;\overset{\;}{↔}\;{\mathrm{Si}}_{3}N_{4}+6\mathrm{CO}$$

During sintering, it is more likely that the SiO_2_ is completely consumed in the partial oxidation of CNTs and CO is no longer formed due to the limited reactant oxygen. Total disappearance of CNTs may happen during the sintering process because of the oxidation of CNTs. During the sintering, the degradation of CNTs takes place in the chemical reactions [64,65].

## 5. The Effects of CNTs Addition on Microstructure Development

The microstructure characteristics after the sintering route of the CNT-reinforced silicon nitride composites are extremely important, because these determine the final mechanical, functional and tribological properties of the composites.

**Figure 5:**
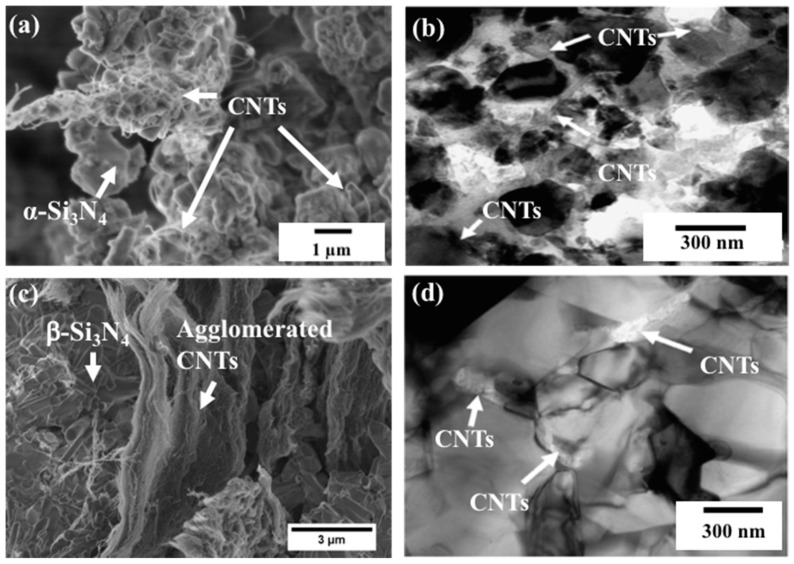
SEM and TEM images of CNTs dispersed in silicon nitride in powders and sintered silicon nitride: (**a**) the network of multi-walled carbon nanotubes (MWCNTs) around the α-Si_3_N_4_ particles (3 wt.% MWCNTs + α-Si_3_N_4_ starting powder with sintering additives Al_2_O_3_, Y_2_O_3_ and PEG); (**b**) bright field TEM images of starting powder α-Si_3_N_4_ with 3 wt.% CNTs prepared by attrition milling, arrows indicate the presence of CNTs between α-Si_3_N_4_ grains [45]; (**c**) in the SEM image, MWCNTs are located in β-Si_3_N_4_ grains of 3 wt.% MWCNTs + Si_3_N_4_ composites. (**d**) Cross-section bright field TEM image shows the CNTs located in porosities and intergranular locations of β-Si_3_N_4_ grains [45]. (Figure 5b–d) Reproduced from Refs. [45] with permission from Elsevier.

Tapasztó et al. [58] studied the dispersibility of the CNTs and multi-layered graphene (MLG) and their impact on the mechanical properties of the silicon nitride composite under the same experimental conditions. Based on the results of scanning electron microscopy (SEM) and small angle neutron scattering (SANS), they concluded that graphene was more efficiently dispersed in the matrix than the carbon nanotubes [58]. The better dispersion of graphene resulted in 10–50% more enhanced mechanical properties as compared to the carbon nanotube ones. Figure 5b–d show the TEM images of CNTs dispersion in silicon nitride before and after sintering [45]. In our experimental work, the MWCNTs made a network around the α-Si_3_N_4_ grains in SEM images of starting powders (Figure 5a). The fractography of fractured surfaces revealed that the agglomerated CNTs were located at the porous locations and the intergranular places of β-Si_3_N_4_ grains (Figure 5c).

As uniform dispersion can transfer the load efficiently and equally, the dispersion has been considered as an important factor in developing composites with better properties. The following novel techniques, such as highly efficient ultrasonic homogenization, high attrition milling, functionalization of CNTs and colloidal processing, have been applied for enhancing the homogeneity of dispersion [15,66,67,68]. Ultrasonic agitation has been proven to be effective in addressing the dispersion problem in the composite [18]. Balázsi et al. found that an increase in sonication time resulted in a better homogeneity [18].

α to β Phase transformation depends on the sintering temperature, holding time and pressure. CNTs do not have a significant effect on the α to β transformation in Si_3_N_4_. Balazsi et al. [15] prepared the silicon nitride composite reinforced with CNTs by using hot isostatic pressing and studied the effect of CNTs on the structural and mechanical properties of the silicon nitride composite. They found the complete α to β transformation in Si_3_N_4_ in the presence of CNTs [15]. The deterioration of CNTs was observed during HIP, which might be caused by the reaction between CNTs and oxygen which evolved the CO and CO_2_ gases [15].

Balazsi et al. [49] observed the presence of both α and β phases in the Si_3_N_4_ + CNT composites which were produced by SPS at 1500 °C for 3 min [49]. This incomplete α to β transformation is due to the low temperature, lower holding time and applied pressure [49]. There is no role of CNTs in incomplete α to β transformation in silicon nitride. In the case of HIP, the complete α to β transformation was observed at 1700 °C for 2 h [49]. Balazsi et al. [14,18] reported the effect of an addition of CNTs on the grain structure of silicon nitride. CNTs provide the crystallization sites for the nucleation and growth of β grains of Si_3_N_4_ [14,69]. They found the MWCNTs in the middle of silicon nitride grains which served as a seed for crystal growth. A. Kovalcıkova et al. [48] reported similar morphological results of silicon nitride composites with the addition of 1 and 3 wt.% CNTs. They found the MWCNTs at intergranular places in the form of agglomeration and had good contact with the silicon nitride grains. The microstructure was mainly composed of β-Si_3_N_4_ grains and no effect of MWCNTs was observed on the α to β transformation. Poor dispersion of MWCNTs was found, which led to lower density (from 3.28 to 2.65 g/cm^3^) with an addition of 3 wt.% CNTs in the matrix.

Highly homogenous CNT-reinforced Si_3_N_4_ can be developed by the in situ growing of nanotubes in the powder by the cobalt catalyst-assisted chemical vapor deposition (CVD) method [68]. Better mechanical properties were achieved after the uniform dispersion of CNTs in the silicon nitride matrix. The growth of in situ CNTs by chemical vapor deposition (CVD) seems efficient and easier than the conventional preparation methods.

The presented results show that morphological characteristics of Si_3_N_4_ grains have a significant influence on the final properties of the composite. The effect of CNTs addition on morphology of Si_3_N_4_ grains has not been widely reported in the literature. However, Pasupuleti et al. [44] reported the effect of CNT addition on Si_3_N_4_ grain structure. They observed the refinement and acicularity of grains with the addition of CNTs. This phenomenon can be explained as the dispersed CNTs provide the nucleation sites for the β grains of Si_3_N_4_. Miranzo et al. [70] also observed the grain refinement with the addition of CNTs in composites prepared by SPS, but they attributed this refinement to an incomplete sintering process. Gonzalez et al. [68] prepared Si_3_N_4_ nanocomposites with 12 vol.% and 22 vol.% in situ grown CNTs, and further densified them using the spark plasma sintering technique. In situ grown CNT composites are materials where the CNTs are synthesized as a reinforcing phase within the matrix during composite development. Gonzalez et al. [68] developed in situ grown CNT composites by mixing the ceramic powder in methanol with cobalt (II) nitrate hexahydrate (Co(NO_3_)_2_·6H_2_O), used as a catalyst precursor, and sonicated for 15 min. For the CNT synthesis, the powder mixture was placed in a tube furnace and the CVD process was carried out at 750 °C for 15 min via thermal decomposition of the acetylene precursor using H_2_ as the process gas. The grain refinement was observed in 12 vol.% in situ grown CNTs + Si_3_N_4_ composites. This trend did not continue for the in situ 22 vol.% CNT-reinforced Si_3_N_4_. This behavior could be explained by Sudre et al.’s model [71]. The inclusions were involved in the composite during the in situ growth of CNTs. According to Sudre et al.’s model [71], these inclusions develop a compressive hydrostatic stress field around themselves during the sintering process and this stress field depends on the volume fraction and aspect ratio of the inclusions. These regions densify faster and become susceptible to grain growth, forming a rigid network that constrains the adjacent porous area, which will be subjected to tensile stresses that promote the de-sintering phenomena in that region. Consequently, both fine grain porous areas and dense areas with large grain growth are developed in the material.

## 6. The Effect of CNTs on Mechanical Properties

### 6.1. Hardness

**Figure 6:**
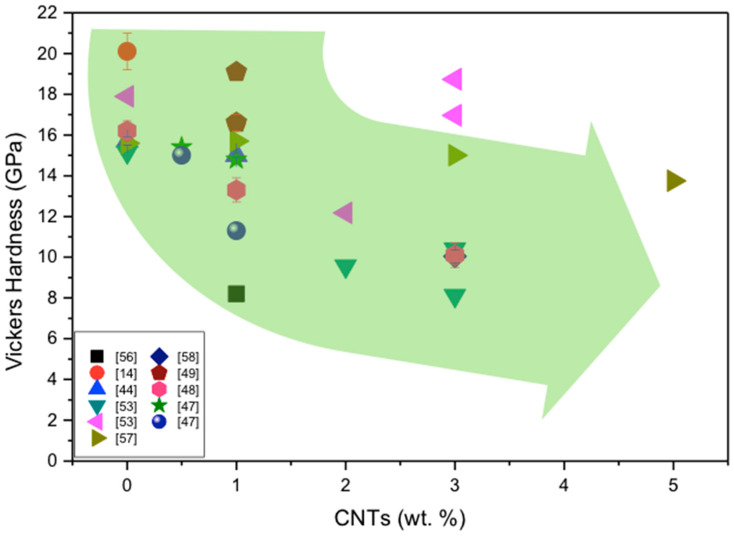
Vickers micro-hardness of CNT-reinforced silicon nitride composites according to the results of different investigations. The green highlighted arrow indicates the decreasing tendency of Vickers hardness of Si_3_N_4_ composites with increasing CNTs content. The details concerning the processing route of the preparation of investigated composites are illustrated in Table 2.

There is not an easy way to compare the hardness of CNT-reinforced Si_3_N_4_ composites prepared and tested by different researchers for two main reasons; even though the composites are processed with the same/similar chemical compositions, the processing routes were more or less different, and the hardness measurement as regards the indentation load was in many cases different. The collected hardness results from the literature are illustrated in Figure 6 and Table 2.

The hardness of monolithic silicon nitride is different due to the different processing route, changing from approximately 14.5 to 20 GPa. The hardness of the composites changes from approximately 8 to 19 GPa depending on content of CNTs and processing route.

According to the results, the hardness values are in a strong relationship with the values of densities.

It has been observed by many researchers that the addition of CNTs induces porosity in the system which degrades the hardness of the composites. Sun et al. also reported that the residual porosity in the composite is responsible for the lower hardness [72]. Similarly, Tian et al. [57] found this behavior in CNT-reinforced Si_3_N_4_ composites and observed the decrease in hardness with the increase in CNT content. Based on the literature studies, we are able to conclude that the hardness of Si_3_N_4_ + CNT composites decreased with the addition of CNTs. This behavior is due to the porosity in the composites which was induced by the CNTs and was not eliminated during the sintering process. As an exception, Tapaszto et al. [53] reported a maximum Vickers hardness value of 18.73 GPa with the addition of 3 wt.% MWCNTs. This, with 3 wt.% MWCNT-reinforced silicon nitride composite, was prepared by SPS with mainly α-Si_3_N_4_, which is harder than the β-Si_3_N_4_ and resulted in a high hardness of the composite [53]. This is an example that high hardness for silicon nitride + CNT composites can be reached by the optimized processing route, which results in high density/low porosity and required microstructural phases-high level of α-Si_3_N_4_.

Figure 6 shows the Vickers hardness of CNT-reinforced silicon nitride composites according to the results of different investigations. The highlighted arrow indicates the wide scatter in the published results and the decreasing tendency of Vickers hardness of Si_3_N_4_ composites with increasing CNT content. The details concerning the processing route of the preparation of investigated composites are illustrated in Table 2. The data are from the literature with volume percent (vol.%) of CNTs converted into weight percent (wt.%) similarly, as in all figures.

### 6.2. Flexural Strength

Similarly, as in the case of hardness, the influence of the CNTs addition on the bending strength values is also not evident and easy to describe, in spite of the fact that the researchers used similar testing methods in the form of a three–point bending test. Balazsi et al. [15] prepared 1 wt.% MWCNT-reinforced Si_3_N_4_ composite by HIP with two different holding times and pressures. They observed a 15–37% improvement in bending strength of the composite in the longer holding time and higher pressure during sintering [15]. They found enhanced strength after increasing the holding time from 1 to 3 h and N_2_ pressure from 2 to 20 MPa. The amount of CNTs should be optimized to achieve the improved mechanical properties. Balazsi et al. [49] developed the silicon nitride composite with 1 wt.% of MWCNTs and the bending strength was found to be higher than that of silicon nitride without MWCNTs. The increase in bending strength is attributed to the high apparent density of the composite [49]. By pulling out, the MWCNTs’ strengthening mechanism was observed in the composite [49]. Yoshio et al. reported that bead milling results in well-pulverized agglomerates of CNTs, uniformly dispersed in ethanol. and in such a way prepared Si_3_N_4_ + CNT ceramics, and the bending strength was improved [60].

Matsuoka et al. reported that bead milling also improved the bending strength of CNT-dispersed Si_3_N_4_ ceramics compared with ball-milled samples at all firing temperatures they applied [47]. They found that the bending strength of a bead-milled composite was as high as the monolithic Si_3_N_4_ ceramics without CNT addition.

**Figure 7:**
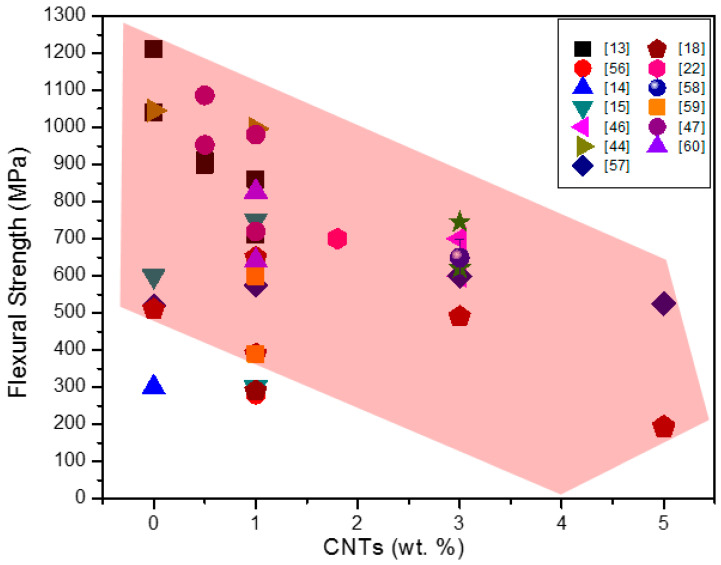
Influence of CNT addition on the flexural strength of CNT-reinforced silicon nitride composites. The results are based on 3-point bending test except the source [44] which is based on 4-point bending strength. The highlighted arrow indicates the decreasing tendency of flexural strength of Si_3_N_4_ composites with increasing CNT content. The processing route for the preparation of investigated materials is illustrated in Table 3.

Selected flexural strength values of Si_3_N_4_ + CNT composites from the literature are presented in Table 3. Figure 7 compares the flexural strength of composites with respect to different contents of CNTs. Here, this flexural strength is based on the three-point bending test, except for one result. The strength values of the composites are very different, even at similar wt.% of CNTs, changing from approximately 200 to 1000 MPa, with the highest values being around 800–900 MPa for the composites with 0.5–1.0 wt.% of CNTs.

To conclude, we can say that inhomogeneous dispersion and agglomeration of CNTs and porosity induced by CNTs result in the degradation in flexural strength of these composites. (Figure 7, Table 3).

#### Fracture Origins

During the bending strength test of advanced ceramics, the fracture origins are usually processing flaws or microstructure imperfections such as pores, non-densified areas, clusters of reinforcement particles, impurities, etc. These kinds of defects are called technological defects, which arise during the processing of composites. Other sources of fracture origin are surface defects which originate from machining or handling the specimens [73,74,75]. These defects during the bending test act as a fracture origin and decrease the strength values according to the character, size and location of the fracture origins.

As it was described before, the problem with the dispersion of CNTs in the silicon nitride matrix often results in the formation of agglomerates and porosity during the sintering process in silicon nitride composites. These defects can cause fractures in the Si_3_N_4_ + CNT composites during the bending tests, as was found by different researchers [18].

With the aim to investigate the effect of powder preparation on the strength of the CNT-dispersed Si_3_N_4_ ceramics, the origins of fractures in these ceramics after the bending test were investigated by Matsuoka et al. [47]. They found that the fracture origin in the composite prepared by ball milling was a large agglomerate of CNTs with a diameter of over 30 m, which probably resulted from insufficient dispersion of the CNTs in the silicon nitride matrix. On the other hand, a fracture origin of the Si_3_N_4_ + CNTs ceramics prepared by bead milling was a pore or a region of insufficient densification, with significantly smaller size than the agglomerate of CNTs in the ball-milled sample.

**Figure 8:**
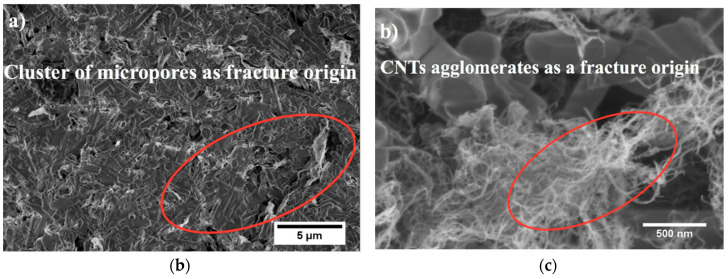
Fracture origins of 3 wt.% MWCNT-reinforced Si_3_N_4_: (**a**) area with micropores (**b**) large agglomerate of MWCNTs. (Authors’work)

Similarly, as in Matsukoa et al. [47], other researchers also found CNT agglomeration and area with micropores, which caused crack initiation during the bending test of the composites (Figure 8). The CNTs agglomerates are weaker zones in the structure which are susceptible to crack initiation and significantly decrease the strength values.

In structural ceramics, characteristic regions are usually around the fracture origins, with such types as mirror, mist and hackle. The mirror is a flat area surrounding the fracture origin, mist is a circle around the outer region of the mirror and the hackle area with ridges is around the mirror and mist region [73]. To the best of our knowledge, such characteristic regions (mirror, mist and hackle) in Si_3_N_4_ + CNT composites have not yet been reported in the literature, and are probably connected with the relatively low bending strength values of the composites.

### 6.3. Fracture Toughness

The fracture toughness of a material defines its ability to resist a fracture. There are several methods to measure the fracture toughness of advanced ceramics, such as single-edged pre-cracked beam (SEPB), chevron notched beam (CNB), surface crack in flexure (SCF) and single-edged V-notched beam (SEVNB). There is also a nonconventional method for measuring the fracture toughness of ceramics based on indentation tests— the so-called indentation fracture toughness method, used mainly during material development and not for reliability prediction. According to the literature results, the fracture toughness of all Si_3_N_4_ + CNT composites was measured mainly by the Vickers indentation fracture (IF) method.

Several researchers reported the enhancement of fracture toughness of Si_3_N_4_ with the addition of CNTs, which was attributed to the toughening mechanism by the CNTs [44,47,53,57]. Some researchers also reported a decrease in fracture toughness of silicon nitride with the addition of CNTs [48,52]. Kovalcıkova et al. [48] reported the decrease in hardness and toughness of silicon nitride composite due to the high level of porosity which was introduced by the addition of MWCNTs. So far, Matsuoka et al. [47] have also reported the highest value of fracture toughness (8.6 MPa·m^1/2^) of 1 wt.% MWCNT-reinforced silicon nitride composite.

As we mentioned, mainly the indentation fracture toughness method has been used for the measurement of the fracture toughness for silicon nitride + CNT composites.

**Figure 9:**
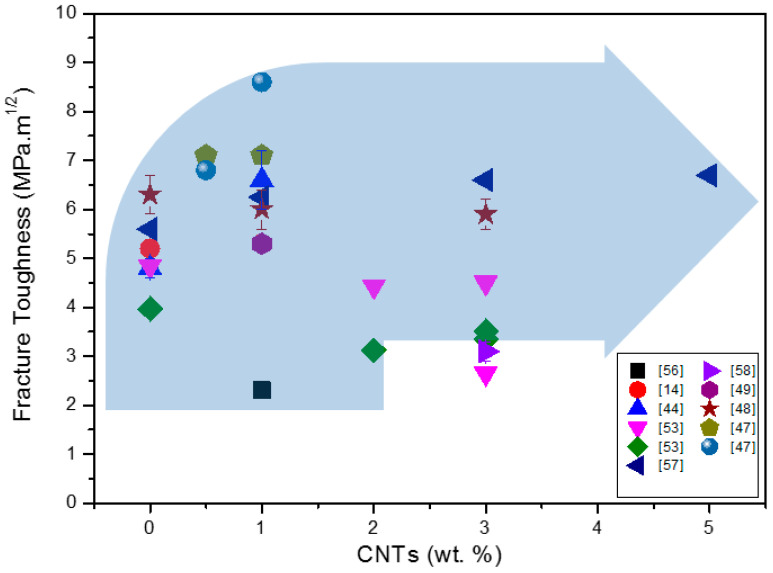
Fracture toughness of CNT-reinforced Si_3_N_4_ composite measured by indentation fracture techniques. The blue highlighted arrow indicates the increasing tendency of fracture toughness of Si_3_N_4_ composites with increasing CNTs content. The processing routes for the preparation of investigated materials are illustrated in Table 4.

Figure 9 shows the fracture toughness behavior of silicon nitride composites with the addition of CNTs.

**Figure 10:**
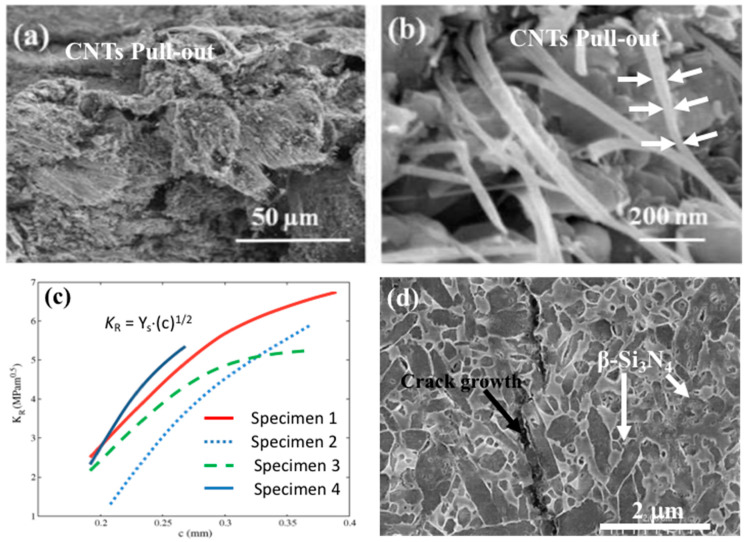
Toughening mechanism in Si_3_N_4_ + CNT composites. (**a**) SEM image of fractured surface of Si_3_N_4_ + CNTs [61]; (**b**) enlarged SEM image of (**a**) revealing the CNTs pulling out from Si_3_N_4_ ceramics, the diameter of CNTs also decreased due to the pulling out and this is an example of good bonding between CNTs and grains [61]; (**c**) crack growth resistance as a function of crack length “c” in CNT-reinforced silicon nitride composites [44]; (**d**) crack propagation is visible in a Si_3_N_4_ + CNT composite [44]. Reproduced from Refs. 44 and 61 with permission from Elsevier.

Pasupuleti et al. were probably the first to perform R-curve measurements on rectangular bars with dimensions of 50 mm length, 4 mm width, and 3 mm depth with an indentation crack on the tensile surface created by 196 N load [44]. The sample was loaded in a four-point bending mode (inner span of 20 mm and outer span of 40 mm) by a servo-hydraulic machine under displacement control with a crosshead speed of 0.05 mm/min. Plots of the crack growth resistance, K_R_, versus the half surface crack length, *c*, were produced to reveal the R-curve characteristics of the systems. The crack growth resistance, K_R_, is the maximum applied stress intensity factor at the tip of the indentation crack for each loading–unloading cycle, see Figure 10. Silicon nitrides with CNT additions show a characteristic toughening (R-curve) behavior with a sharp rise in the crack growth resistance with crack length followed by a plateau.

Table 4 presents the fracture toughness values reported in the literature along with the sintering parameters and density. Only selected results are shown in Table 4.

As is visible, the scatter in measured values is very high and the fracture toughness values for the systems with similar CNT contents are significantly different, probably caused by different processing routes and microstructures of the systems, but also with measuring methods. It seems to be true that an addition of CNTs to approximately 1 wt.% results in the improvement of fracture toughness; however, a higher amount of the CNT addition does not contribute to the further improvement of the fracture toughness of the composites.

#### Toughening Mechanisms

The fracture toughness of brittle ceramics can be increased by the addition of a proper second phase as a reinforcement which initiates different reinforcing mechanisms during the crack propagation and increases the fracture toughness.

As above it was mentioned to improve the toughness of Si_3_N_4_, two strategies can be adopted (i.e., in situ toughening and ex situ toughening). The in situ toughening mechanism is promoted by the elongated β–Si_3_N_4_ grains which act as a self-reinforcing agent in the matrix [43,76,77,78]. Ex situ toughening mechanisms are connected with the introduced secondary phases to the system in the form of fibers nanotubes or grapheme platelets [79,80,81,82,83]. In Si_3_N_4_ composites, introducing a secondary phase can create crack bridging reinforcing mechanisms in the form of crack deflection, pull-out, and frictional/mechanical interlocking, etc.

Carbon nanotubes as toughening agents in the ceramic materials have been widely investigated in the last decade [14,15,18,44,50,68,84,85,86,87,88]. In an ideal case, when a crack propagates in the matrix and confronts CNTs, the CNTs can deflect the crack at a certain angle. In this way, the crack-propagating fracture energy is consumed, the crack-propagating rate is reduced, and the toughness of the composite is improved. However, crack deflection by CNTs is not common because CNTs are not robust enough to deflect the crack. In another case, when a crack propagates and encounters the CNTs, crack bridging (the CNTs connect the crack surfaces by its two ends) or pull-out of CNTs will occur. In this way, the crack-propagating fracture energy is consumed and delays the fracture, which eventually enhances the fracture toughness. Crack bridging through the pulling-out of CNTs is common in Si_3_N_4_ composites. Carbon nanotubes can toughen the silicon nitride composites, but the toughening mechanism depends on many factors, such as the amount of CNTs, dispersion, shape, aspect ratio, contact area of CNTs with matrix grains, porosity and other processing-related defects.

Xia et al. [87] carried out research on the toughening behavior of CNT-reinforced ceramic composites for the first time. They demonstrated crack deflection, crack bridging and fiber pull-out in the CNT-reinforced composites. Tatami et al. [22] and Balazsi et al. [14] developed CNT-reinforced silicon nitride composites and they reported improvements in strength, stiffness and toughness with the addition of CNTs. All these mechanisms delay the fracture in the composites. Several challenges must be dealt with to develop these toughening mechanisms in the silicon nitride composites. Effective load transfer plays a role in enhancing the toughness and it depends on the interfacial strength between CNTs and silicon nitride grains. Without the optimum interfacial strength, the effective load transfer is not possible, which leads to the diminishing of crack-bridging and pulling-out mechanisms on the fracture surface. The damage to CNTs during the sintering is another problem which has been reported by researchers [15]. CNTs can be preserved during the sintering process by adopting the novel processing technique spark plasma sintering (SPS) [14,89].

As the result of the active toughening mechanisms, the crack growth resistance (fracture toughness) rises with the crack extension, which can be illustrated by a so-called R–curve, which is a plot of resistance to fracture versus crack extension. Pasupuleti et al. [44] measured the R-curve behavior in 1 wt.% CNT-reinforced silicon nitride composite and observed a sharp rise in the crack growth with the crack length (Figure 10c,d). Crack bridging and pulling-out of CNTs were the major causes of this behavior. According to the reported results, the crack resistance “*K_R_*” increases with the crack length “c” in CNT-reinforced Si_3_N_4_ composite (Figure 10c) [44]. This refers to a situation where fracture toughness increases with the increasing size of the crack in ceramics. It shows that CNTs induced the toughening mechanisms by crack bridging and pulling-out in silicon nitride-based ceramics. Ge et al. [61] also observed the pulling-out of CNTs in the SEM image of a fractured surface of a Si_3_N_4_ + CNT composite (Figure 10a,b). The diameter of CNTs decreased from 50 to 10 nm in the direction of pulling and this reveals the good agreement between CNTs and silicon nitride grains [61]. When the composite fractured, the CNT bundles were fine-drawn and pulled out when 10–20 μm in length [61].

**Figure 11:**
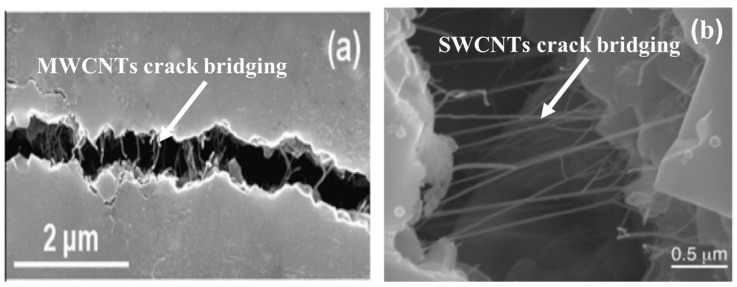
Crack bridging by CNTs in Si_3_N_4_ + CNT systems, [50,68]. Reproduced from Refs. 50 and 68 with permission from John Wiley and Sons and Elsevier, respectively. (**a**) crack bridging by MWCNTs [68]; (**b**) crack bridging by SWCNTs [50].

The crack bridging was also observed as a toughening mechanism on the fracture line, as is illustrated for CNTs in Figure 11 [50,68].

Toughening is also influenced by the interface between the CNT and silicon nitride grains. It was evident from earlier studies that for ideal toughening in fiber-reinforced ceramic composites, the nature of the interface between the fiber and the matrix is very important. The interface must be of sufficiently low toughness to debond upon impingement of the matrix crack and must subsequently not slide too easily or with too much difficulty. Due to easy inter-wall sliding, MWCNTs can alter the toughening behavior. This behavior affects the load transfer from outer to inner walls and consequently denounces the strength and toughness.

Another aspect of enhancing the fracture toughness is the direction of the CNTs in the silicon nitride matrix. The perpendicular direction of CNT fibers to the crack direction is helpful in enhancing the toughness. The perpendicular direction of CNTs toward crack propagation deflects the crack and delays the fracture. The degradation of CNTs was observed due to the defects by several researchers and these defects may break down the CNTs in the sites when stress is applied, and these defects can be the origin of the fracture [57]. It seems that a difference in the thermal coefficient of expansion (CTE) between CNT and Si_3_N_4_ can improve the fracture toughness. During the cooling process, stresses are induced which initiate an internal crack to propagate toward the CNTs and the crack is deflected by the CNTs, which is the contribution to enhance the fracture toughness [57].

It is possible to say that in Si_3_N_4_ + CNT composites, the carbon nanotubes improve the fracture toughness through the toughening mechanisms such as small length scale crack deflection and crack bridging connected with the pull-out of CNTs from the ceramic matrix. Advanced processing routes—green processing methods, colloidal processing, and optimized sintering with SPS, etc.—result in such a mechanism during the fracture process and, in the fracture toughness, an improvement up to 8.5 MPa·m^1/2^ can be observed. It is also visible that the increasing of CNT content above 1.5 wt.% does not result in fracture toughness improvement.

### 6.4. Tribological Properties

Tribology is the study of interacting surfaces of two bodies. Friction and wear happen as the result of two surfaces which are mechanically in contact and slide against each other. This deals with adhesion, friction, wear and lubrication in all contacting areas. The solid knowledge of tribology helps to improve the service life, safety, and reliability of interacting machine components and yields substantial economic benefits. There are two aspects of tribology: the first is science which deals with the basic mechanism, and the second is technology which deals with design, manufacture and maintenance.

The common test geometries used to study wear are pin-on-flat, four-ball, ring-on-flat, pin and V-block, and rolling/sliding disk contact [90].

Tribological properties of carbon based nanostructured reinforced Si_3_N_4_ composites have been widely investigated during the last decade [24,51,68,76,77,78,79,80,81,82,83,91,92,93,94].

**Figure 12:**
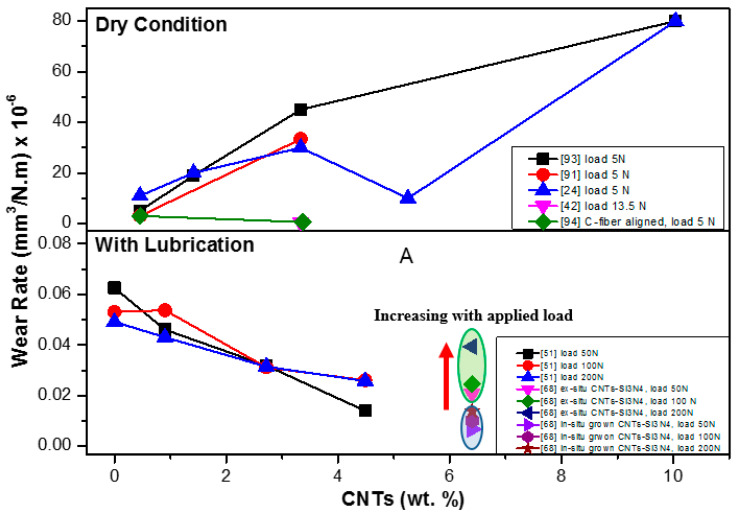
Influence of the CNTs addition on the wear rate of Si_3_N_4_ + CNT composites reported by different researchers.

**Figure 13:**
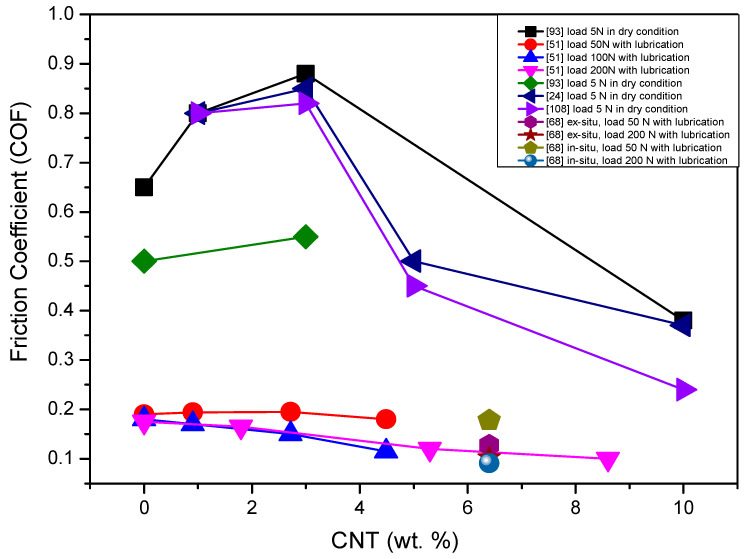
Influence of the CNTs addition on the coefficient of friction during the tribological test of Si_3_N_4_ + CNT composites reported by different researchers.

Figure 12 and Figure 13 illustrate the influence of CNT addition on wear rate and the coefficient of friction during the tribological tests of CNT-reinforced silicon nitrides tested up to now.

Koszor et al. reported that the friction coefficient decreased with the addition of 3 wt.% MWCNTs in Si_3_N_4_ composite [91]. The wear rate of monolithic silicon nitride composite was reported to be 3.02 × 10^−6^ mm^3^/N·m and the addition of 3 wt.% MWCNTs reduced the wear to 3.34 × 10^−5^ mm^3^/N·m [91].

Gonzalez-Julian et al. [51] found the better tribological properties of 8.6 vol.% MWCNT-reinforced Si_3_N_4_ composites than the monolithic Si_3_N_4_ composite under the load in isooctane lubrication condition. The better tribological properties were attributed to the dense homogeneity of CNTs and the extra effect of lubrication by CNTs. It was observed that Si_3_N_4_ + MWCNT composites showed 40% lower friction coefficient and 80% lower wear rates than that of the monolithic silicon nitride materials.

Wang et al. [92] proposed that wear performance can be enhanced by the redistribution of stresses under load contact and that CNTs can play a vital role in this regard. CNTs must bend, twist and make a network around the Si_3_N_4_ grains to sustain and distribute the stresses developed by contact load. It was observed that uniform dispersion and good interfacial bonding of CNTs with grains of silicon nitride enhance the wear resistance.

Balko et al. [93] prepared the silicon nitride composite with 1, 3, 5 and 10 wt.% of multi-walled carbon nanotubes (MWCNTs) at 1700 °C by the HIP sintering technique. They performed the tribological tests on these composites using a ball on desk configuration in dry conditions. Notably, 1 and 3 wt.% of MWCNTs did not display any significant role in decreasing the coefficient of friction and wear rate, but the MWCNTs higher than 5 wt.% had a positive effect in reducing the wear rate and coefficient of friction (COF). In addition, 10 wt.% MWCNT-reinforced Si_3_N_4_ reduced the coefficient of friction (COF) by 46% compared to that of 1 wt.%.

Recently, a highly wear-resistant Si_3_N_4_ + MWCNT composite was developed with uniform homogeneity of MWCNTs which were grown by in situ CNTs using the cobalt catalyst-assisted CVD method [68]. This in situ composite was 87% and 65% more wear resistant than the monolithic Si_3_N_4_ and ex situ Si_3_N_4_ composite, respectively. Friction coefficients of monolithic Si_3_N_4_, ex situ Si_3_N_4_ and in situ Si_3_N_4_ are 0.19, 0.175 and 0.13 under 50 N load, respectively.

In monolithic silicon nitride composites, the general wear mechanism is that the grains are detached from the surface during the sliding and these grains cause the abrasion and pronounce the effect of wearing. In general, wear debris is formed by the action of the micro-abrasion mechanism, being compacted during the motion of the sliding pairs. If CNTs are present in the debris wear, then the debris wear serves as lubrication and overcomes friction. One of the examples was observed by Gonzalez-Julian et al. [68] in in situ CNTs + Si_3_N_4_ composites, the debris areas appeared well adhered to the surface which protected it against wear [68].

In Figure 12 and Figure 13, the very strong difference in the wear behavior of the composites tested under dry and lubricated conditions is visible. According to the results, the CNTs’ addition to the silicon nitride has a very positive effect on the wear rate and coefficient of friction. It is evident that the nanotubes provide an important lubricating effect in the tribo-system, especially at high loads, through the development of CNT-based layers and have an important role in improving the wear resistance of the composites.

## 7. Thermal and Functional Properties of Si_3_N_4_ + CNT Composites

### 7.1. Thermal Shock Resistance

Successful application of advanced materials at high temperatures requires the development of systems with good thermal shock resistance. Pettersson et al. [95] proved that a material with better thermal shock resistance can absorb residual stresses at a higher load than that of a material with poorer thermal shock resistance. Good thermal shock resistance enables ceramic materials to sustain without failure in a rapid heating and cooling environment. Measuring the thermal shock resistance of ceramic composites is crucial prior to applying them under high temperature loads. Microstructure, tensile strength, fracture toughness, Biot modulus, Young’s modulus and thermal expansion coefficient are influential parameters in the thermal shock resistance of any material [95].

Kovalcıkova et al. [48] studied the effect of the addition of MWCNTs on the thermal shock resistance of silicon nitride composites and they found a positive effect of a limited addition of MWCNTs. They reported that the addition of 1 wt.% CNTs enhanced the thermal shock resistance of the composite. However, by adding 3 wt.% of CNTs in Si_3_N_4_, the thermal shock resistance of the composite decreased. There could be two possible reasons for lower thermal shock resistance [48]. One of them is lower strength values of the samples due to the addition of CNTs. Another reason is a difference in coefficients of thermal expansion (CTE) of silicon nitride (3 × 10^−6^ K^−1^) and MWCNTs (1.6–2.6 × 10^−5^ K^−1^). The difference in CTEs of Si_3_N_4_ and CNTs exert internal stresses upon heating and cooling, which lead to crack extension. In general, materials with a high flexural strength, R-curve, high Young’s modulus and high fracture toughness exhibit the highest thermal shock resistance.

### 7.2. Thermal Conductivity

Most of the work reported a decrease in thermal conductivity of silicon nitride composites with the addition of carbon nanotubes [55,96,97]. The α/β phase ratio is important in describing the thermal conductivity of silicon nitride composites. Miranzo et al. [97] reported the thermal properties as a function of β phase in the composite. Meanwhile, a decrease in thermal conductivity was reported with the addition of CNTs. The decrease is associated with the tube–tube junctions, chirality, temperature, diameter and intrinsic defects within the carbon nanotubes [97,98,99]. The strained sigma bonds govern the thermal conductivity of CNTs. Zhang et al. [100] studied the thermal conductivities of three types of single-wall carbon nanotubes using the homogeneous non-equilibrium Green–Kubo method based on the Brenner potential. They found that the sigma bonds along the circumference are intensely strained in armchair and chiral CNTs, but the sigma bond is minimally strained in the zigzag type of CNTs. The ultra-strain along the circumference limits the phonon mean free path because of scattering, which lowers the thermal conductivity. Therefore, the thermal conductivity of zigzag is higher than that of armchair and chiral nanotubes. CNTs have a tendency of bending and twisting in the composites and this behavior induces intrinsic defects. These intrinsic defects act as a barrier to thermal transport in ceramic composites. Although it is predicted that carbon nanotubes have a high longitudinal thermal conductivity (5800 Wm^−1^ K^−1^) at room temperature, it is very difficult to realize this effect in a composite. The CNTs in the composites are bent, entangled and clustered, which induces the interfacial and intrinsic defects which offer resistance in heat flow and reduce the thermal conductivity.

**Figure 14:**
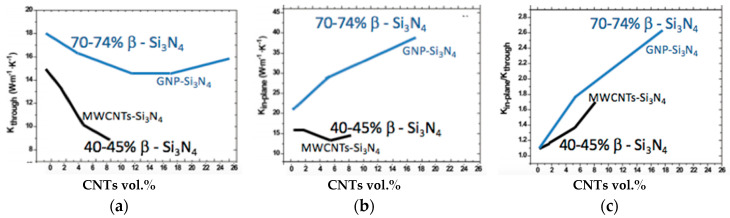
Thermal conductivity of CNT- and graphene nanoplatelets (GNP)-reinforced Si_3_N_4_: (**a**) through-thickness; (**b**) in-plane; and K_in-plane_/K_through-thickness_ ratio, (**c**) silion nitride composites with MWCNTs and GNP as a function of carbon nanostructures, based on [94].

Miranzo et al. [97] fabricated Si_3_N_4_ with the concentration of MWCNTs ranging from 0.9 to 8.6 vol.% and studied their anisotropic thermal conductivities. They reported a decrease of 33% in the thermal conductivity through thickness with an addition of 5.3 vol.% MWCNTs. A slight increase in thermal conductivity in the direction of in-plane was reported. This enhancement of thermal conductivity is attributed to the preferred orientation of the CNTs which offer less resistance in the in-plane direction (Figure 14). TEM images revealed that MWCNTS are bent and twisted at the grain boundaries in these materials. Pettes and Shi also reported that the thermal conductivity of MWCNTs depends upon the defects within the nanotubes [101].

Corral et al. [96] also reported a 62% decrease in thermal conductivity of Si_3_N_4_ with the addition of 2 vol.% of SWCNTs. Koszor et al. [91] claimed the enhancement of thermal conductivity of Si_3_N_4_ with the integration of MWCNTs but without considering the effects of β phase in the composite.

According to Miranzo et al. [97], β phase content has a positive effect in enhancing the thermal conductivity. To our understanding, the enhancement of thermal conductivity of the composite claimed by Koszor et al. [91] was likely due to the presence of β phase, not of the CNTs.

Osendi et al. [55] also reported reduction in thermal conductivity and thermal diffusivity of Si_3_N_4_ with the addition of 5.3 vol.% CNTs.

### 7.3. Electrical Conductivity

Silicon nitride was reported as an insulator material which has electrical resistivity >1014 Ω·cm at room temperature and >106 Ω·cm at 1200 °C [102]. Si_3_N_4_ can be employed in the power generation industry (micro-turbines) and telecommunication industry (micro-electro-mechanical systems, MEMS) but its insulating behavior limits its use in this sector. For use in the electrical sector, changing the Si_3_N_4_ from an insulating to a conducting material is therefore required.

Carbon nanotubes are highly electrically conductive due to their carbon structure, but the electrical conductance depends on the chirality of CNTs. Carbon nanotubes are oriented in the form of hexagons and the types of orientation of hexagons determine the electrical properties of CNTs. There are three basic types of orientation: armchair, zigzag and chiral. These three orientations are described above in Figure 2. Ceramic materials have poor electrical conductivity because they lack free electrons in the valence shell. To enhance the electrical conductivity of ceramics, CNTs are one of the best candidates. In the literature, the enhancement of electrical conductivity by the addition of CNTs in ceramic composites has been reported [13,20,22,47,48,60,61,91,96,103,104,105,106,107,108].

Balázsi et al. [18] found a positive effect on the electrical conductivity of the silicon nitride with the addition of 0–5 wt.% MWCNTs. They observed that the electrical behavior of the composite was changed from insulator to conductor with the addition of MWCNTs. However, the level of conductance was dependent on the type, size, amount, shape and distribution of MWCNTs in the composite [18].

**Figure 15:**
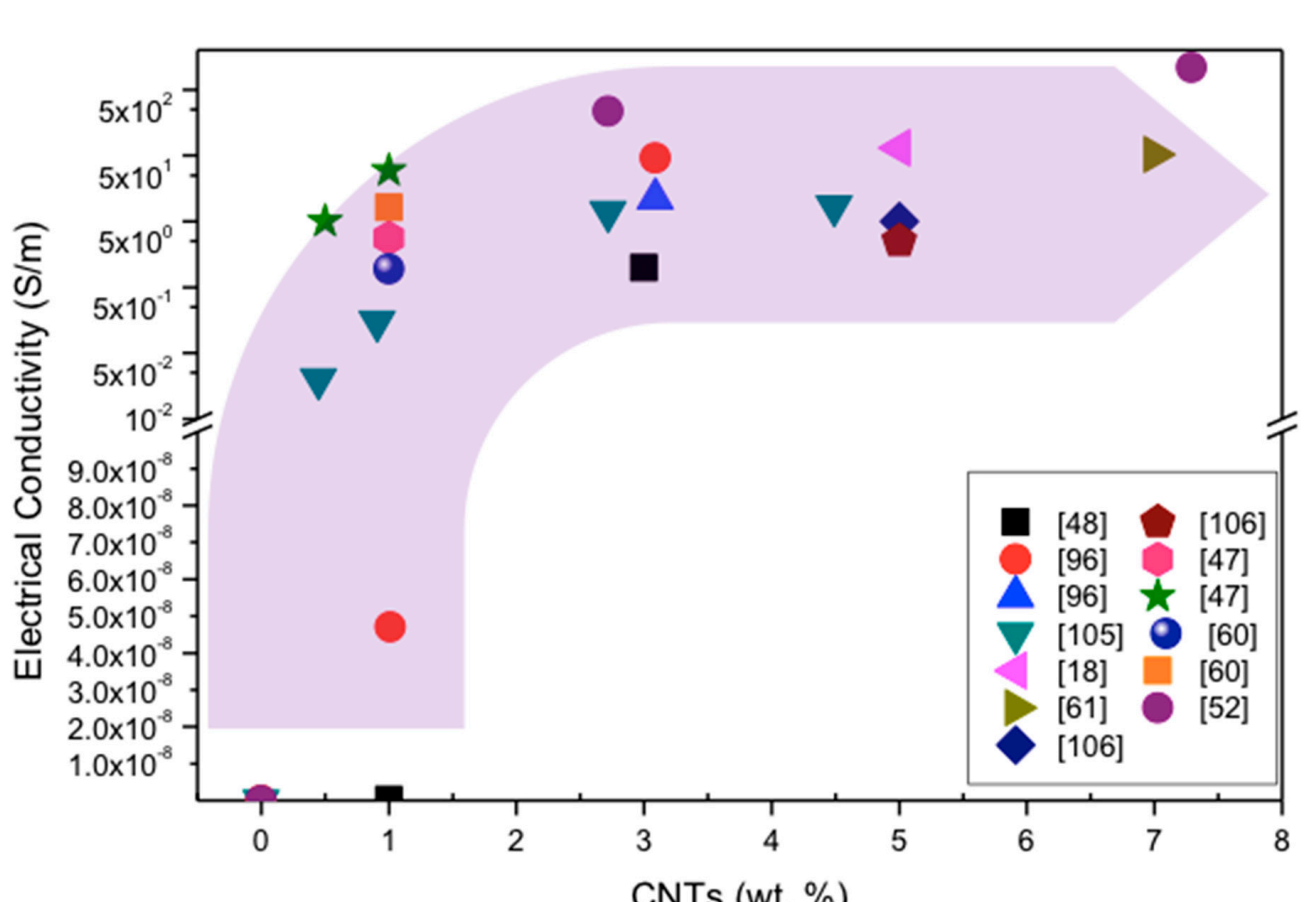
Electrical conductivity of CNT-reinforced silicon nitride composites. The processing routes for the preparation of investigated materials are illustrated in Table 5. The purple highlighted arrow indicates the increasing tendency of electrical conductivity of Si_3_N_4_ composites with increasing CNTs content in the matrix.

Some typical values of electrical conductivity of CNT-reinforced Si_3_N_4_ are given in Figure 15. Most of the researchers reported in weight percent (wt.%) units and a few researchers reported CNTs by volume percent (vol.%) units.

Kovalcikova et al. [48] also reported the significant improvement in electrical conductivity of 2 S/m of silicon nitride with the addition of 3 wt.% MWCNTs.

Hvizdos et al. [24] reported the increment in electrical conductivity of several ceramic materials with the addition of CNTs and carbon nanofibers (CNFs). The addition of 10 wt.% CNTs/CNFs increased the electrical conductivity of silicon nitride from 1 × 10^−13^ to 1 × 102 S/m [24].

Ge et al. reported the electrical conductivity (1.03 × 10^2^ S/m) of CNT fiber-reinforced silicon nitride composites which was attributed to the internal CNT network [61].

CNTs should develop the electrical conductance path in the ceramic. Porosities are induced by densification inhibition and break the electrical conductance path in the composites. It was also observed that the clustering of CNTs did not play a role in increasing the electrical conductivity because it breaks the conductance path in ceramics.

In 2013, M. Belmonte et al. [52] reported the so far highest electrical conductivity value of 2174 S/m in nitrogen-doped CNT-reinforced Si_3_N_4_ composites. CNTs were synthesized by chemical vapor deposition (CVD) at 800 °C in argon (Ar) gas atmosphere. For the source of carbon and nitrogen, an aerosol solution containing 6 wt.% FeCP_2_ and 94 wt.% C_7_H_9_N was used, respectively [52,109]. Carbon nanotubes were treated with H_2_O_2_ in sonication for 30 h to disperse and isolate the nanotubes from the bundles. This dispersion procedure was effective in functionalizing the CNTs’ surfaces and promoted the agreement between CNTs and Si_3_N_4_ grains, which enhanced the electrical conductivity of the composite.

Some typical values of electrical conductivity of CNT-reinforced Si_3_N_4_ are given in Table 5. The increase in electrical conductivity of Si_3_N_4_ composites can be observed with the addition of CNTs (Table 5).

The results show that an optimized processing route for CNT preparation and preparation of Si_3_N_4_ + CNT composites results in high electrical conductivity values, higher than 2174 S/m.

## 8. Summary and Future Challenges

In this overview, the results published to date concerning the development, processing, microstructure characteristics and properties of silicon nitride/carbon nanotubes composites are summarized.

1. The main processing routes and microstructure characterization techniques during the development of the Si_3_N_4_ + CNT composites are the following:
  - Four different types of CNTs have been used as additives to the silicon nitride matrix; commercially available single-walled and multi-walled carbon nanotubes with different geometries and impurity contents, modified CNTs by replacing carbon by nitrogen atoms and CNTs prepared by in situ synthesis.
  - The most often used sintering additives are Y_2_O_3_ and Al_2_O_3_, but AlN, HfO_2_, TiO_2_ have been used as well.
  - Ball milling, bead milling, ultrasonic bath-stirring, attritor milling and colloidal processing have been used with the aim of preparing highly dispersed aqueous composite suspensions containing carbon nanotubes before the sintering.
  - HP, HIP, GPS, SPS, microwave sintering and their combination were applied for the sintering of Si_3_N_4_ + CNT composites.
  - The most common microstructure characterization usually involves pictures of low-magnification fracture surfaces acquired by either SEM or optical microscopy, field emission scanning electron microscopy (FESEM), TEM, HRTEM, energy filtered-TEM (EFTEM) and small angle neutron scattering (SANS). Raman spectroscopy has been performed with the aim of quantitatively describing the CNT network throughout Si_3_N_4_ matrix.
2. The influence of processing routes on the microstructure characteristic revealed that:
  - CNT-dispersed Si_3_N_4_ ceramics fabricated with new additives (HfO_2_) for lower-temperature densification and using bead milling for homogeneous dispersion of CNTs results in high-density composites without a cluster of CNTs and significantly improved final properties.
  - The SPS technique has a strong potential for designing Si_3_N_4_ materials with tailored microstructures and appears to be the best tool to achieve full densification of new ceramic composites containing graphite structures such as CNTs, avoiding their degradation.
  - Si_3_N_4_ + CNT composites sintered by the spark plasma sintering method mainly consist of α-Si_3_N_4_ and so are harder and stiffer. The composites prepared by hot isostatic pressing are characterized by β-Si_3_N_4_ grains and provide a tougher matrix.
  - Based on the experiments, it is clear that the CNT positions and distributions play critical roles in the ability of this additive to improve the densification and mechanical properties. Homogeneously distributed CNTs containing composites are potentially useful for many structural applications, but agglomerations or weak reinforcing phases and matrix grain interfaces need to be avoided.
  - Nitrogen-doped CNTs (CNx) bundles sonicated in hydrogen peroxide (H_2_O_2_) with increased surface functionalization allowed the complete dispersion of the bundles and resulted in perfectly dispersed individual nanotubes and an absence of impurity particles. The fast heating rates of the SPS technique avoided the nanotube degradation during the sintering of dense Si_3_N_4_ + CNT composites with a good dispersion of the CNx within the ceramic matrix, even for the material with high CNx content (13.6 vol.%).
  - The CVD processing for in situ CNT growth ensures a more uniform dispersion in the matrix than traditional ex situ CNT mixing methods without the formation of bundles seen with traditional ex situ mixing of CNTs in silicon nitride-based composites. Compared to ex situ nanocomposites, in situ ones exhibit not only much higher CNTs dispersion but a stronger nanotube/matrix interface that leads to an improved mechanical performance
3. The effect of the CNT addition on the mechanical properties—hardness, bending strength and fracture toughness—and tribological characteristics—wear rate and coefficient of friction—can be summarized as follows:
  - It is not easy to compare the published mechanical and tribological properties reported by different authors, as the results of experiments are realized using different methods, e.g., in hardness measurement, different applied loads or in fracture toughness measurement by indentation methods or SEPB method, etc.
  - The hardness of the composites was usually lower in comparison to the hardness of silicon nitride; however, in some cases, the composites with a low volume fraction of CNTs show a similar hardness compared to the monolithic system thanks to the homogenously distributed CNTs, small grain-sized matrix with α–silicon nitride.
  - The bending strength of the Si_3_N_4_ + CNT composites decrease with the increasing volume fraction of the CNTs due to the present structural defects, acting as a fracture origin and resulting in lower bending strength values. However, improved processing in the form of bead milling can eliminate/limit the presence of strength decreasing defects and grant a bending strength value for the composite similar to the values of the strength of the monolithic Si_3_N_4_ ceramics without CNTs, with values up to 1000 MPa.
  - The reported fracture toughness of the Si_3_N_4_ + CNT composites is the highest usually at a CNT content of approximately 1 wt.%. It seems that the fracture toughness of the Si_3_N_4_ + CNT composite can systematically increase from 5.3 to 8.5 MPa·m^1/2^ by increasing the SWCNT loading from 0 to 2.0 vol.% with the application of highly controlled green processing methods, colloidal processing, and optimized sintered microstructures with SPS. Microcantilever experiments provide direct evidence of CNTs’ toughening effect in Si_3_N_4_ ceramics and the measured *K_R_* (a) shows an increase in fracture resistance with crack extension.
  - The nanocomposites containing up to 5 wt.% of CNTs, processed using sonicating and mechanical stirring procedures in ethanol media and further densification by SPS decreased the friction coefficient and enhanced the wear resistance by about 40% and 80%, respectively, as compared with the monolithic ones. In situ reinforced composites with even higher CNT content exhibit excellent tribological behavior, being 87% and 65% more wear resistant than monolithic Si_3_N_4_ and ex situ nanocomposites, respectively, and also leading to the lowest friction at high contact pressures. According to the results, the nanotubes provide an important lubricating effect in the tribo-system, especially at high loads, though the development of CNT-based layers also has an important role in improving the wear resistance of the composites.
4. The influence of the CNTs addition on the thermal and functional properties of the composites shows that:
  - CNT-dispersed Si_3_N_4_ ceramics with a high density and good electrical conductivity were developed using novel sintering aids. The electrical conductivity has a high value of 30 S/m for the system prepared by GPS/HIP, and 79 S/m prepared by HP. Novel highly electrically conductive Si_3_N_4_ + CNT SPS sintered nanocomposites containing up to 13.6 vol.% of nitrogen-doped multi-walled carbon nanotubes reached an electrical conductivity of 2174 S/m, which is the highest value reported hitherto for carbon nanotubes/Si_3_N_4_ nanocomposites.
  - A reduced thermal conductivity for Si_3_N_4_ + CNT composites has been reported over the monolithic silicon nitride.
5. Further Challenges
  Further progress is expected in the development of Si_3_N_4_ + CNT composites with the aim to:
  - solve the problem of difficulties relating to dispersing CNTs mainly with an increasing concentration of nanotubes by the help of advanced processing such as colloidal processing, etc. This will help not only in the elimination/limitation of strength-decreasing defects in the composites, but also in increasing the number of active nanotubes in the toughening process and an increased number of constituents for increasing the tribological and functional properties as well.
  - realize an effective CNT reinforcement strategy while optimizing the CNT/matrix interface in such a way as to have the adhesion between the nanotube and the matrix be not so strong as to introduce nanotube failure before debonding, but to have the adhesion be not so weak that the frictional resistance to sliding is minimal.
  - make advances in improving the properties of modified CNT fillers and in the field of in situ reinforced composites with the aim to offer processing of Si_3_N_4_ + CNT composites with improved functional, tribological and mechanical properties.
  - improve the most promising processing methods such as aqueous colloidal processing, ultrasonication, bead milling, improved SPS, electric field-assisted pressure-less sintering, usually named flash sintering, etc.
  - introduce new characterization and testing methods in the area of Raman spectroscopy, focused ion–beam (FIB) technique, microcantilever technique for fracture toughness testing, etc.
  - design new systems in the form of CNT-concentrated, functionally graded and layered CNT–ceramic composites, etc., in combination with other carbon-based fillers as graphene platelets which would surely offer multi-functional properties for challenging functional, bio-medical and structural applications.
  - Improve the applications of CNT ceramic matrix nanocomposites such as: load-bearing structural parts, wear or friction surfaces, medical devices and implants, automotive, aerospace, power generation applications, tool and die materials, and military field applications.

## Figures and Tables

**Table 1:** Comparison of properties of CNT and other materials. Reproduced from [38].

Material	Density (g/cm^3^)	Tensile Strength (GPa)	Stiffness (GPa)
CNTs	1.3–2	10–60	1000
Wood	0.6	0.008	16
Steel	7.8	0.4	208
Carbon Fiber	1.7–2.2	1.7–3.3	200–960
Epoxy	1.25	0.005	3.5

**Table 2:** Vickers hardness of CNT-reinforced silicon nitride composites reported in the literature measured using different applied loads.

Si_3_N_4_ + CNTs	Milling Parameter	Sintering Parameters	Sintering Additives	Theoretical/Apparent Density (g/cm^3^)	Load (N)	Vickers Hardness (GPa)	Ref.
1 wt.% MWCNTs	Planetary	GRF/1000~1450 °C/40 h + 550 °C/2 h	MgO, Al_2_O_3_, SiO_2_	89.4%	-	8.2	[56]
1 wt.% MWCNTs	Ball/3 h	SPS/1500 °C/5 min/50 MPa	Al_2_O_3_, Y_2_O_3_	3.17 g/cm^3^	10	16.6 ± 0.4	[14]
1 wt.% MWCNTs	Ball/24 h	HP/1750 °C /1 h/30 MPa	Al_2_O_3_, Y_2_O_3_, ZrO_2_	98.7%	98	15.0 ± 0.1	[44]
3 wt.% MWCNTs	Attritor/5 h	HIP/1700 °C/ 3 h/20 MPa	Al_2_O_3_, Y_2_O_3_	-	98	10.41	[53]
3 wt.% MWCNTs	Attritor/5 h	SPS/1650 °C/3–5 min/50 MPa	Al_2_O_3_, Y_2_O_3_	-	98	18.73	[53]
3 wt.% SWCNTs	Attritor/5 h	SPS/1650°C/3–5 min/50 MPa	Al_2_O_3_, Y_2_O_3_	-	98	16.97	[53]
5 wt.% MWCNTs	Ball/16 h	HP/1700 °C/1 h/ 30 MPa	Al_2_O_3_, Y_2_O_3_	-	29	~13.75	[57]
3 wt.% MWCNTs	Attritor/3 h	HIP/1700 °C/0/20 MPa	Y_2_O_3_, Al_2_O_3_	2.7 g/cm^3^	98	10.04 ± 0.3	[58]
1 wt.% MWCNTs	Ball/3 h	SPS/1500 °C/5 min/100 MPa	Y_2_O_3_, Al_2_O_3_	3.17 g/cm^3^	-	16.6	[49]
1 wt.% MWCNTs	Ball/3 h	SPS/1500 °C/3 min/50 MPa	Y_2_O_3_, Al_2_O_3_	3.19 g/cm^3^	-	19.1	[49]
3 wt.% MWCNTs	Ball/3 h	HIP/1700 °C/3 h/20 MPa	Y_2_O_3_, Al_2_O_3_	2.65 g/cm^3^	98	10.1 ± 0.6	[48]
1 wt.% MWCNTs	Bead/2 h	GPS/1600–1750 °C/2 h + HIP/1700°/1 h/100 MPa	Y_2_O_3_, Al_2_O_3_, AlN. HfO_2_, TiO_2_	99.6%	98	14.8	[47]
1 wt.% MWCNTs	Ball/24 h	GPS/1600–1750 °C/2 h + HIP/1700°/1 h/100 MPa	Y_2_O_3_, Al_2_O_3_, AlN. HfO_2_, TiO_2_	93.5%	98	11.3	[47]
1 vol.% SWCNTs	Ball/12 h	SPS/1600 °C/3 min	CTAB *	95.4%	2.45	17.6	[50]
6 vol.% SWCNTs	Ball/12 h	SPS/1600 °C/3 min	CTAB *	91.0%	2.45	10.7	[50]
1.8 vol.% MWCNTs	Ball/24 h	SPS/1550 °C/5 min/50 MPa	Y_2_O_3_, Al_2_O_3_	3.22 g/cm^3^	98	16.4 ± 0.1	[55]
5.3 vol.% MWCNTs	Ball/24 h	SPS/1600 °C/5 min/50 MPa	Y_2_O_3_, Al_2_O_3_	3.19 g/cm^3^	98	12.6 ± 0.3	[55]
3 wt.% MWCNTs	Ball/4 h	HIP/1700 °C/3 h/20 MPa	Y_2_O_3_, Al_2_O_3_	93.4%	10	4.88 ± 0.2	[42]

* Cetrimonium bromide.

**Table 3:** Flexural strength of Si_3_N_4_ + CNT composites prepared by different processing routes.

Si_3_N_4_ + CNTs	Milling Type Parameters	Sintering Parameters	Sintering Additives	Theoretical Density/Apparent Density	Flexural Strength (MPa)	Ref.
0.5 wt.% MWCNTs	Ball/48 h	GPS/1750 °C/2 h + HIP/1700 °C/1 h/100 MPa	HfO_2_, TiO_2_, Y_2_O_3_, Al_2_O_3_, AlN	>97%	900	[13]
1 wt.% MWCNTs	Ball/48 h	GPS/1750 °C/2 h + HIP/1700 °C/1 h/100 MPa	HfO_2_, TiO_2_, Y_2_O_3_, Al_2_O_3_, AlN	>97%	860	[13]
1 wt.% MWCNTs	Planetary	GRF/1000 ~ 1450 °C/40 h + 550 °C/2 h	MgO, Al_2_O_3_, SiO_2_	89.4% / 2.83 g/cm^3^	280	[56]
1 wt.% MWCNTs	Planetary	HIP/1700 °C/h/2 MPa	Al_2_O_3_, Y_2_O_3_	2.5 g/cm^3^	~ 295	[14]
1 wt.% MWCNTs	Ball/24 h	HIP/1700 °C/ h/2 MPa	Al_2_O_3_, Y_2_O_3_	3.007 g/cm^3^	750	[15]
3 wt.% MWCNTs	Attritor/5 h	GPS/1700 °C/3 h/20 MPa	Al_2_O_3_, Y_2_O_3_	3.33 g/cm^3^	~ 745	[46]
1 wt.% MWCNTs	Ball/24 h	HP/1750 °C–HP/1 h/30 MPa	Al_2_O_3_, Y_2_O_3_, ZrO_2_	98.7%	996 ± 25 (4 pt.)	[44]
5 wt.% MWCNTs	Ball/16 h	HP/1750 °C/1 h/30 MPa	Al_2_O_3_, Y_2_O_3_	-	525	[57]
1 wt.% MWCNTs	Ball/3 h	HIP/1700 °C/3 h/20 MPa	Al_2_O_3_, Y_2_O_3_	3.8 g/cm^3^	650	[18]
3 wt.% MWCNTs	Ball/3 h	HIP/1700 °C/3 h/20 MPa	Al_2_O_3_, Y_2_O_3_	2.7 g/cm^3^	490	[18]
1.8 wt.% MWCNTs	Ball/48 h	Fired/1800 °C/2 h + HIP/1700 °C/1 h/100 MPa	Y_2_O_3_, Al_2_O_3_, TiO_2_, AlN	~97%	700	[22]
12 wt.% MWCNTs	Ball/48 h	HP/1800 °C/2 h/30 MPa	Y_2_O_3_, Al_2_O_3_, TiO_2_, AlN	~92%	580	[22]
3 wt.% MWCNTs	Attritor/5 h	GPS/1700 °C/3 h/20 MPa	Y_2_O_3_, Al_2_O_3_	3.330 g/cm^3^	745	[46]
3 wt.% MWCNTs	Attritor/3 h	HIP/1700°C/0/20MPa	Y_2_O_3_, Al_2_O_3_	2.7 g/cm^3^	649 ± 50	[58]
1 wt.% MWCNTs	Ball/3 h	HIP/1700 °C/3 h/20 MPa.	Y_2_O_3_, Al_2_O_3_	3.08 g/cm^3^	~600	[59]
0.5 wt.% MWCNTs	Bead/2 h	GPS/1600–1750 °C/2 h + HIP/1700 °C/1 h/100 MPa	Y_2_O_3_, Al_2_O_3_, AlN, HfO_2_, TiO_2_	99.8%	1086	[47]
1 wt.% MWCNTs	Bead/2 h	GPS/1600–1750 °C/2 h + HIP/1700 °C/1 h/100 MPa	Y_2_O_3_, Al_2_O_3_, AlN, HfO_2_, TiO_2_	99.6%	~980	[47]
1 wt.% MWCNTs	Bead/4 h	GPS/1700 °C/2 h + HIP/1700 °C/1 h/100 MPa	Y_2_O_3_, Al_2_O_3_, AlN, HfO_2_, TiO_2_	98.4%	827	[60]
1 wt.% MWCNTs	Ball/24 h	GPS/1700 °C/2 h + HIP/1700 °C/1 h/100 MPa	Y_2_O_3_, Al_2_O_3_, AlN, HfO_2_, TiO_2_	96.2%	642	[60]
3 wt.% MWCNTs	Ball/4 h	HIP/1700 °C/3 h/20 MPa	Y_2_O_3_, Al_2_O_3_	93.4%	313.4	[42]

**Table 4:** Fracture toughness of CNT-reinforced silicon nitride composites reported by different researchers.

Si_3_N_4_ + CNTs	Milling Parameter	Sintering Parameters	Sintering Additives	Theoretical Density/Apparent Density	Indentation Fracture Toughness (MPa·m^1/2^)	Ref.
1 wt.% MWCNTs	Planetary	GRF/1000~1450 °C/40 h + 550 °C/2 h	MgO, Al_2_O_3_, SiO_2_	2.83 g/cm^3^	2.3	[56]
1 wt.% MWCNTs	Ball/3 h	SPS/1500 °C/5 min/50 MPa	Al_2_O_3_, Y_2_O_3_	3.17 g/cm^3^	5.3	[14]
1 wt.% MWCNTs	Ball/24 h	HP/1750 °C/1 h/30 MPa	Al_2_O_3_, Y_2_O_3_, ZrO_2_	3.22 g/cm^3^	6.6 ± 0.6	[44]
3 wt.% SWCNTs	Attritor/5 h	HIP/1700 °C/3 h/20 MPa	Al_2_O_3_, Y_2_O_3_	-	2.65	[53]
3 wt.% MWCNTs	Attritor/5 h	HIP/1700 °C/3 h/20 MPa	Al_2_O_3_, Y_2_O_3_	-	4.5	[53]
3 wt.% SWCNTs	Attritor/5 h	SPS/1650 °C/5 min/100 MPa	Al_2_O_3_, Y_2_O_3_	-	3.51	[53]
3 wt.% MWCNTs	Attritor/5 h	SPS/1650 °C/5 min/100 MPa	Al_2_O_3_, Y_2_O_3_	-	3.35	[53]
5 wt.% MWCNTs	Ball/16 h	HP/1700 °C/1 h/30 MPa	Al_2_O_3_, Y_2_O_3_	-	6.7	[57]
3 wt.% MWCNTs	Attritor/3 h	HIP/1700 °C/0/20 MPa	Y_2_O_3_, Al_2_O_3_	2.7 g/cm^3^	3.1 ± 0.2	[58]
1 wt.% MWCNTs	Attritor/3 h	SPS/1500 °C/5 min/100 MPa	Y_2_O_3_, Al_2_O_3_	3.17 g/cm^3^	5.3	[49]
3 wt.% MWCNTs	Ball/3 h	HIP/1700 °C/3 h/20 MPa	Y_2_O_3_, Al_2_O_3_	2.65 g/cm^3^	5.9 ± 0.3	[48]
1 wt.% MWCNTs	Bead/2 h	GPS/1600–1750 °C/2 h + HIP/1700 °C/1 h/100 MPa	Y_2_O_3_, Al_2_O_3_, AlN. HfO_2_, TiO_2_	99.6%	7.1	[47]
1 wt.% MWCNTs	Ball/24 h	GPS/1600–1750 °C/2 h + HIP/1700 °C/1 h/100 MPa	Y_2_O_3_, Al_2_O_3_, AlN. HfO_2_, TiO_2_	93.5%	8.6	[47]
5.3 vol.% MWCNTs	Attritor/2 h	SPS/1585–1600 °C/5 min/50 MPa	Y_2_O_3_, Al_2_O_3_	3.15 g/cm^3^	4.8 ± 0.1	[52]
2 vol.% SWCNTs	Ball/12 h	SPS/1700 °C/3 min/-	CTAB *	94.2%	8.78 ± 0	[50]
2 vol.% SWCNTs	Ball/12 h	SPS/1600 °C/0 min/-	CTAB *	91.0%	8.48 ± 0.0	[50]
8.6 vol.% CNT	Ultrasonicstir	SPS/1585 °C/5 min/50 MPa	Al_2_O_3_, Y_2_O_3_	3.12 g/cm^3^	4.2 ± 0.1	[51]
5.3 vol.% MWCNTs	Ball/24 h	SPS/1600 °C/5 min/50 MPa	Y_2_O_3_, Al_2_O_3_	3.19 g/cm^3^	4.2 ± 0.1	[55]

* Cetrimonium bromide.

**Table 5:** Electrical conductivity of CNT-reinforced Si_3_N_4_ composites.

Si_3_N_4_ + CNTs.	Theoretical Density/Apparent Density	Sintering Parameters	Sintering Additives	Electrical Conductivity (S/m)	Ref.
1 wt.% MWCNTs	3.14 g/cm^3^	HIP/1700 °C/3 h/20 MPA	Al_2_O_3_, Y_2_O_3_	257 × 10^−12^	[48]
3 wt.% MWCNT	2.65 g/cm^3^	HIP/1700 °C/3 h/20 MPa	Al_2_O_3_, Y_2_O_3_	1.98	
6 vol.% SWNTs	91%	SPS/600 °C/ 3 min/-	Y_2_O_3_, MgO, Al_2_O_3_	91.91	[96]
6 vol.% SWNTs	76.5%	SPS/1300 °C /3 min/-	Y_2_O_3_, MgO, Al_2_O_3_	22.01	[96]
5.3 vol.% MWCNTs	99%	SPS/1585 °C/ 5 min/-	-	14	[105]
8.6 vol.% MWCNTs	99%	SPS/1585 °C/ 5 min/-	-	17	[105]
5 wt.% MWCNTs	2.3 g/cm^3^	HIP/1700 °C/-/ 2 MPa	AlN, Al_2_O_3_, Y_2_O_3_	130	[18]
7 wt.% MWCNTFs		RBSN/1450 °C/2 h/ 0.08 MPa	-	103	[61]
5 wt.% MWCNTs	-	GPS/1700 °C/-/2 MPa	Y_2_O_3_, Al_2_O_3_	10	[106]
5 wt.% MWCNTs	-	HIP/1700 °C/3 h /20 MPa	Y_2_O_3_, Al_2_O_3_,	5	[106]
0.5 wt.% MWCNTs	99.8%	GPS/1600-1750 °C/2 h + HIP/1700 °C/1 h/100 MPa	Y_2_O_3_, Al_2_O_3_, AlN. HfO_2_, TiO_2_	1.5 × 10^−4^	[47]
1 wt.% MWCNTs	93.5%	GPS/1600–1750 °C/2 h + HIP/1700 °C/1 h/100 MPa	Y_2_O_3_, Al_2_O_3_, AlN. HfO_2_, TiO_2_	59	[47]
1 wt.% MWCNTs	93.5%	HIP/1700 °C/1 h /100 MPa	Y_2_O_3_, Al_2_O_3_, AlN, HfO_2_, TiO_2_	16.3	[60]
5.3 vol.% MWCNTs	3.15 g/cm^3^	SPS/1600 °C/5 min/50 MPa	Y_2_O_3_, Al_2_O_3_	474	[52]
13.6 vol.% CNx	3.03 g/cm^3^	SPS/1600 °C/5 min/50 MPa	Y_2_O_3_, Al_2_O_3_	2174	[52]
0.5 wt.% MWCNTs	-	GPS/1700 °C/2 h/0.9 MPa	TiO_2_, Y_2_O_3_, Al_2_O_3_, AlN	18	[13]
1 wt.% MWCNTs	-	GPS/1700 °C/2 h/0.9 MPa	HfO_2_, Y_2_O_3_, Al_2_O_3_, AlN	49	[13]
12 wt.% MWCNTs	>95%	HP/1800 °C/2 h/30 MPa	Y_2_O_3_, Al_2_O_3_, TiO_2_, AlN	>10^2^	[22]
